# Postoperative recurrence with right cervical lymph node metastasis in hepatocellular carcinoma: a case report

**DOI:** 10.1186/s40792-021-01352-y

**Published:** 2021-12-17

**Authors:** Kiyonori Tanoue, Yota Kawasaki, Yoichi Yamasaki, Satoshi Iino, Masahiko Sakoda, Yuko Mataki, Tetsuya Idichi, Yoshiaki Kita, Yuto Hozaka, Akihiro Nakajo, Takaaki Arigami, Hiroshi Kurahara, Takao Ohtsuka

**Affiliations:** 1 Department of Digestive Surgery, Breast and Thyroid Surgery, Kagoshima University Graduate School of Medical and Dental Sciences, 8-35-1 Sakuragaoka, Kagoshima, 890-8520 Japan; 2 Department of Digestive Surgery, Kagoshima City Hospital, Kagoshima, Japan; 3 Department of Digestive Surgery, Kagoshima Kouseiren Hospital, Kagoshima, Japan; 4 Department of Onco-biological Surgery, Kagoshima University Graduate School of Medical and Dental Sciences, Kagoshima, Japan

**Keywords:** Hepatocellular carcinoma, Recurrence, Extrahepatic metastasis, Cervical lymph node metastasis

## Abstract

**Background:**

Hepatocellular carcinoma (HCC) patients with metastases to the cervical lymph nodes are extremely rare, and its clinical course is characterized by rapidly progressive disease. Hence, there have been no reports of metastatic cervical lymph node recurrence indicated after a long postoperative surveillance period.

**Case presentation:**

The patient was a 63-year-old male who underwent right hepatectomy for HCC of the right upper lobe. Three years after resection, metastatic lymph node recurrence was detected in the subdiaphragm, superior mediastinum, and right cervical lymph nodes. The patient underwent excisional biopsy of the cervical lymph node, followed by molecular-targeted therapy and radiation therapy. Lenvatinib reduced the size of all metastatic lymph nodes and the patient survived for a relatively long period of 43 months after the recurrence was detected.

**Conclusions:**

After resection of HCC in the right upper lobe, there is the possibility of metastatic lymph node recurrence in unusual sites, including the cervical region, and lenvatinib may be effective in those recurrences.

## Background

Hepatocellular carcinoma (HCC) constitutes more than 90% of the primary tumors of the liver and is now the fifth most common cancer worldwide [1, 2]. The most common sites of extrahepatic metastasis in HCC are the lungs, brain, adrenal gland, bones, and lymph nodes [3–6], although the incidence of lymphatic metastasis in HCC is far lower than that of hematogenous metastasis [3–5]. In addition, metastasis of HCC to the cervical lymph node has rarely been reported, and its clinical course is characterized by rapidly progressive disease [7, 8]. Here, we report a patient with HCC who experienced metastatic right cervical lymph node recurrence after a long postoperative period, and a relatively long survival was obtained by treatment using molecular-targeted therapy.

## Case presentation

**Fig. 1:**
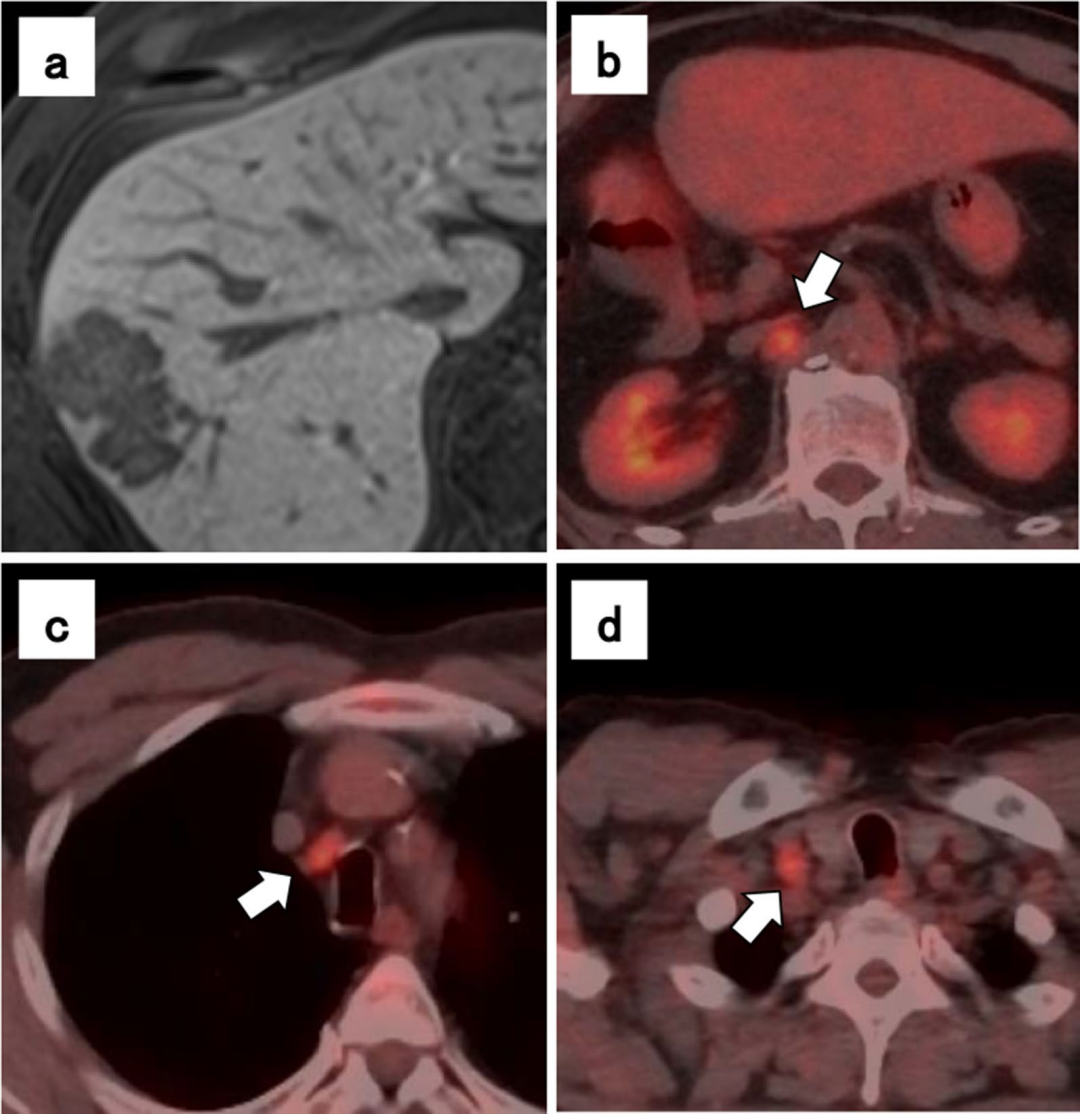
Hepatocellular carcinoma findings on magnetic resonance imaging, and lymph node metastasis on positron emission tomography–computed tomography (PET–CT) imaging. The tumor was located at the border of segments 8 and 7 of the liver (**a**). Lymph node metastasis seen on PET–CT, a subdiaphragmatic lymph node (**b**), an upper mediastinal lymph node (**c**), and a right cervical lymph node (**d**)

**Fig. 2:**
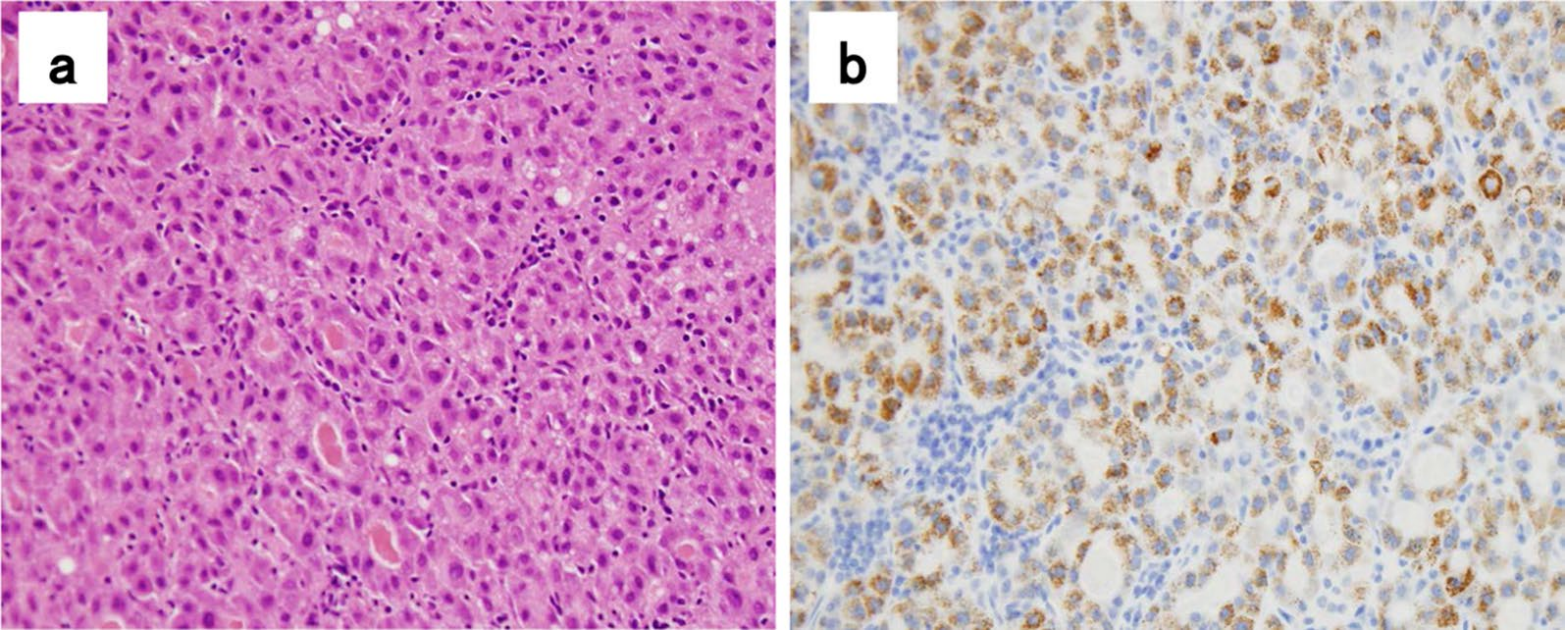
Histological finding on hematoxylin–eosin staining and immunohistochemistry. **a** Cervical lymph node metastasis. **b** Hepatocyte positivity in tumor cells

**Fig. 3:**
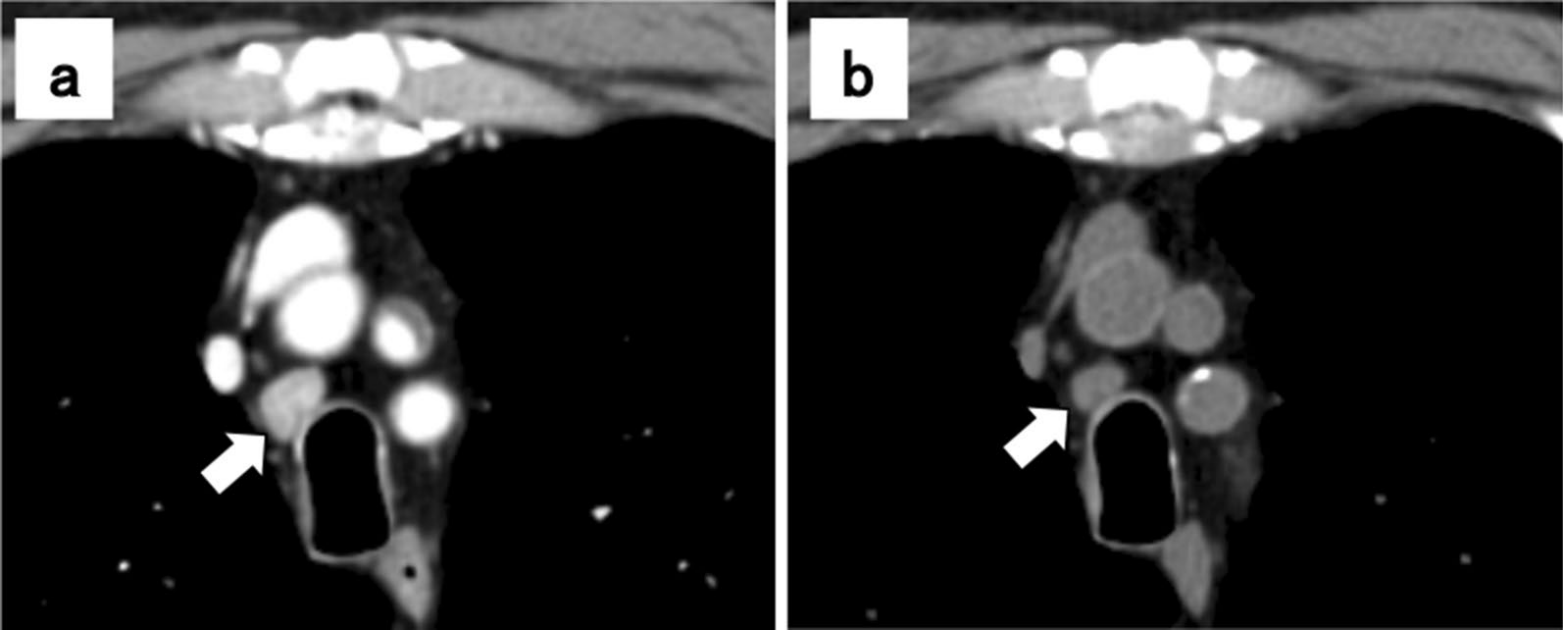
Computed tomography images of the upper mediastinal lymph node metastasis before (**a**) and after 2 months of lenvatinib treatment (**b**)

The patient was a 63-year-old male who underwent right hepatic lobectomy for tumor 5 cm in diameter located at the border of segments 8 and 7 of the liver (Fig. 1a). There were no suspicious findings of extrahepatic metastasis, and pathological examination revealed HCC with moderately differentiated, confluent multinodular type, H2, St-AP 40 mm, pseudoglandular type, e.g., fc(−), fc-inf(−), sf(+), s0, vp1, vv0, va0, b0, sm(−) 30 mm, f1, pT3 (the Japanese classification of primary liver cancer sixth edition). No lymph node metastasis or evidence of intrahepatic metastasis was found. Three years postoperatively, alpha-fetoprotein (preoperative vs postoperative, 656 vs 8.9 ng/ml) was slightly elevated at 36.7 ng/ml in a routine laboratory examination. Due to the elevated alpha-fetoprotein level, abdominal computed tomography (CT) was performed and a recurrent lesion was revealed lymphadenopathy with a tendency to increase in size below the right diaphragm. Positron emission tomography (PET) revealed dense fluorodeoxyglucose uptake in a subdiaphragmatic lymph node (Fig. 1b), an upper mediastinal lymph node (Fig. 1c), and a right cervical lymph node (Fig. 1d). The patient underwent an excisional biopsy of the cervical lymph node, and the pathological findings were compatible with those of metastatic HCC, with swollen nuclei and eosinophilic follicles in tumor cells showing a chordate or alveolar pattern (Fig. 2a, b). Based on these findings, the patient was diagnosed with recurrent HCC characterized by multiple lymph node metastases. The patient was started on sorafenib, a tyrosine kinase inhibitor, and the sizes of metastatic right cervical and mediastinal lymph nodes in imaging studies decreased 2 months later. However, the size of the metastatic subdiaphragmatic lymph node gradually increased. After receiving radiotherapy at a dose of 47.7 Gy for the subdiaphragmatic lymph node, the patient was started on lenvatinib, a tyrosine kinase inhibitor, following which all metastatic lymph nodes reduced in size and maintained for 15 months (Fig. 3a, b). However, because renal function worsened as an adverse effect of lenvatinib treatment, its administration was discontinued. The tumor enlarged rapidly, and the patient died 15 months later. The patient survived 43 months after the detection of recurrence and 79 months after liver resection.

## Discussion

In HCC, hematogenous metastasis is more common than lymph node metastasis. Autopsy studies have showed that the frequency of lymph node metastasis in HCC is 30.3% [9]. However, in patients who underwent hepatic resection, the frequency of lymph node metastasis has been reported to be only 1.0% [10]. Clinically, lymph node metastasis is rare, and distant lymph nodes are rarely involved [4]. The majority of the lymph flow toward the hepatic hilum passes through the hepatoduodenal ligament. In contrast, a small amount of flow is connected to the diaphragm and intrathoracic lymphatic system via the bilateral triangular ligaments [11]. The atypical metastasis to the subdiaphragmatic, superior mediastinal, and cervical lymph nodes seen in this patient may be due to the latter pathway. In fact, it was reported that cervical lymph node metastasis is more frequent in patients with tumors in the right upper lobe [4], which supports our experience. There are few case reports of HCC patients with cervical lymph node metastasis. In total, 11 patients have been reported in 10 case reports. Ten of the 11 patients had cervical lymph node metastasis confirmed at the time of initial diagnosis [7, 8, 12–19]. Because of the rapid disease progression in these patients, there are no reports of patients with resectable disease or metastatic cervical lymph node recurrence after resection. To the best of our knowledge, this is the first report of cervical lymph node metastasis detected a relatively long time after surgery for the primary tumor. Lymph node metastasis commonly occurs in poorly differentiated HCC [4, 20]. Further, high alpha-fetoprotein level (> 10 ng/mL), large tumor size (> 5 cm), and presence of microvascular invasion have been reported as risk factors for lymph node metastasis in HCC [21]. In the postoperative surveillance of patients with tumors located in the right upper lobe of the liver and who have risk factors for lymph node metastasis, as in the present patient, we should consider the possibility of metastatic lymph node recurrence.

Cervical lymph node metastases are found mainly in the left cervical lymph nodes and are thought to be mediated by the thoracic duct. In contrast, there are a few patients reported with metastasis to the right cervical lymph nodes [12, 18, 19]. The presence of chronic liver disease, such as alcoholic hepatitis, accelerates the lymphatic flow and causes a complex bypass of lymphatic flow due to lymphatic vessel occlusion [20]. This change in lymph flow is one of the possible causes of lymph node metastasis to the right cervical lymph nodes. The prognosis for patients with HCC presenting with lymph node metastasis is very poor. Among the case reports of cervical lymph node metastasis in HCC, the median survival in the five reports that clearly described prognosis was 1.5 months (range 1–11 months) [7, 8, 14, 16, 18]. In previous reports, the longest survival time after the detection of cervical lymph node metastasis was 11 months. However, in our patient, the metastatic lymph node size was maintained for a long period with lenvatinib treatment, resulting in a 43-month survival after the diagnosis of recurrence. Lenvatinib is superior to sorafenib as the first-line treatment regimen for unresectable HCC [22] and may be effective in the treatment of metastatic multiple lymph node recurrence involving cervical lymph nodes. This patient was treated with sorafenib as first-line treatment and then lenvatinib after it was covered by Japanese insurance. Renal dysfunction occurred as an adverse effect in this patient, and lenvatinib treatment was discontinued, although the therapeutic effect persisted. Since the size of the metastatic lymph nodes increased rapidly after treatment discontinuation, it was considered important to administer lenvatinib for as long as possible with appropriate dose reduction and drug withdrawal.

If lymph node metastasis is solitary, surgical resection [23] can be considered. In a study on resection in extrahepatic metastases, including lymph node metastases, the presence of two or more extrahepatic lesions was independently correlated with poor survival [24]. This suggests that resection of multiple lesions does not contribute to prolonged survival. In our patient, the recurrence of metastatic lymph nodes was multiple and distant; therefore, we considered that the resection of all metastatic lymph nodes might not have been effective.

## Conclusions

After resection of HCC in the right upper lobe, there is the possibility of metastatic lymph node recurrence in unusual sites, including the cervical region, and lenvatinib may be effective in those recurrences.

## Data Availability

Not applicable.

## Abbreviations

HCC: Hepatocellular carcinoma
CT: Computed tomography
PET: Positron emission tomography

## References

[1] Ioannou GN, Splan MF, Weiss NS, McDonald GB, Beretta L, Lee SP. Incidence and predictors of hepatocellular carcinoma in patients with cirrhosis. Clin Gastroenterol Hepatol. 2007;5(8):938–45, 45 e1–4. 10.1016/j.cgh.2007.02.039 17509946

[2] Ferlay J, Soerjomataram I, Dikshit R, Eser S, Mathers C, Rebelo M (2015). Cancer incidence and mortality worldwide: sources, methods and major patterns in GLOBOCAN 2012. Int J Cancer.

[3] Yuki K, Hirohashi S, Sakamoto M, Kanai T, Shimosato Y. Growth and spread of hepatocellular carcinoma. A review of 240 consecutive autopsy cases. Cancer. 1990;66(10):2174–9. 10.1002/1097-0142(19901115)66:10<2174::aid-cncr2820661022>3.0.co;2-a 2171748

[4] Watanabe J, Nakashima O, Kojiro M (1994). Clinicopathologic study on lymph node metastasis of hepatocellular carcinoma: a retrospective study of 660 consecutive autopsy cases. Jpn J Clin Oncol.

[5] Katyal S, Oliver JH, Peterson MS, Ferris JV, Carr BS, Baron RL (2000). Extrahepatic metastases of hepatocellular carcinoma. Radiology.

[6] Ikai I, Arii S, Okazaki M, Okita K, Omata M, Kojiro M (2007). Report of the 17th nationwide follow-up survey of primary liver cancer in Japan. Hepatol Res.

[7] Liu T, Gao JF, Yi YX, Ding H, Liu W (2013). Misdiagnosis of left supraclavicular lymph node metastasis of hepatocellular carcinoma: a case report. World J Gastroenterol.

[8] Toyoda H, Fukuda Y, Koyama Y, Nishimura D, Hoshino H, Katada N (1996). Case report: multiple systemic lymph node metastases from a small hepatocellular carcinoma. J Gastroenterol Hepatol.

[9] Liver Cancer Study Group of J. Primary liver cancer in Japan. Clinicopathologic features and results of surgical treatment. Ann Surg. 1990;211(3):277–87. PMC1358432 2155591

[10] Kudo M, Izumi N, Kubo S, Kokudo N, Sakamoto M, Shiina S (2020). Report of the 20th nationwide follow-up survey of primary liver cancer in Japan. Hepatol Res.

[11] Magari S (1990). Hepatic lymphatic system: structure and function. J Gastroenterol Hepatol.

[12] Madabhavi I, Patel A, Choudhary M, Anand A, Panchal H, Parikh S (2014). Right cervical lymphadenopathy: a rare presentation of metastatic hepatocellular carcinoma. Gastroenterol Hepatol Bed Bench.

[13] Kobayashi K, Himoto T, Tani J, Miyoshi H, Yoneyama H, Deguchi A (2012). A rare case of hepatocellular carcinoma accompanied by metastasis of a cervical lymph node. Intern Med.

[14] Karaman E, Cansiz H, Acioglu E, Yilmazbayhan D (2007). Primary manifestation of hepatocellular carcinoma as a cervical mass. Kulak Burun Bogaz Ihtis Derg.

[15] Mondal RK, Dutta A, Basu K, Chakraborti S (2005). Virchows node: rare presentation of childhood hepatocellular carcinoma. Indian J Pediatr.

[16] Taniai N, Yoshida H, Mamada Y, Mizuguchi Y, Fujihira T, Akimaru K (2005). A case of recurring hepatocellular carcinoma with a solitary Virchow’s lymph node metastasis. J Nippon Med Sch.

[17] Koklu S, Arhan M, Kuksal A, Ulker A, Temuuin T (2003). An unusual presentation of hepatocellular carcinoma. Int J Gastrointest Cancer.

[18] Thorburn D, Sanai FM, Ghent CN (2003). Hepatocellular carcinoma presenting as right supraclavicular lymphadenopathy. Can J Gastroenterol.

[19] Lau KK, Wong KW, Ho WC, Lai TS, Lee KC, Leung TW (2000). Hepatocellular carcinoma presenting with right supraclavicular lymph node metastasis and superior mediastinal syndrome. Liver.

[20] Abe T, Furuse J, Yoshino M, Kinoshita T, Konishi M, Inoue K (2002). Clinical characteristics of hepatocellular carcinoma with an extensive lymph node metastasis at diagnosis. Am J Clin Oncol.

[21] Lee CW, Chan KM, Lee CF, Yu MC, Lee WC, Wu TJ (2011). Hepatic resection for hepatocellular carcinoma with lymph node metastasis: clinicopathological analysis and survival outcome. Asian J Surg.

[22] Kim S, Kim KH, Kim BK, Park JY, Ahn SH, Kim DY (2021). Lenvatinib is independently associated with the reduced risk of progressive disease when compared with sorafenib in patients with advanced hepatocellular carcinoma. J Gastroenterol Hepatol.

[23] Uenishi T, Hirohashi K, Shuto T, Kubo S, Tanaka H, Sakata C (2000). The clinical significance of lymph node metastases in patients undergoing surgery for hepatocellular carcinoma. Surg Today.

[24] Berger Y, Spivack JH, Heskel M, Aycart SN, Labow DM, Sarpel U (2016). Extrahepatic metastasectomy for hepatocellular carcinoma: predictors of long-term survival. J Surg Oncol.

